# Cannabinoid receptor 2 selective agonist ameliorates adjuvant-induced arthritis by modulating the balance between Treg and Th17 cells

**DOI:** 10.3389/fphar.2025.1532518

**Published:** 2025-01-31

**Authors:** Na Tian, Cui Yang, Yu Du, Miao Chen, Bin Li, Dan Li, Sheng-Ming Dai

**Affiliations:** ^1^ Department of Rheumatology and Immunology, Shanghai Sixth People’s Hospital Affiliated to Shanghai Jiao Tong University School of Medicine, Shanghai, China; ^2^ Center for Immune-Related Diseases at Shanghai Institute of Immunology, Department of Respiratory and Critical Care Medicine of Ruijin Hospital, Department of Thoracic Surgery of Ruijin Hospital, Department of Immunology and Microbiology, Shanghai Jiao Tong University School of Medicine, Shanghai, China; ^3^ Department of Integrated TCM and Western Medicine, Shanghai Skin Disease Hospital, School of Medicine, Tongji University, Shanghai, China; ^4^ Department of Thoracic Surgery, Shanghai Pulmonary Hospital, Tongji University, Shanghai, China; ^5^ Department of Oncology, The First Affiliated Hospital of Anhui Medical University, Hefei, China; ^6^ Department of Hepatobiliary Surgery, The First Affiliated Hospital of Anhui Medical University, Hefei, China

**Keywords:** rheumatoid arthritis, cannabinoid receptor 2, Th17/Treg balance, adjuvant-induced arthritis, cd4+ T cell differentiation

## Abstract

**Background:**

Adjuvant-induced arthritis (AIA) serves as a classic model for rheumatoid arthritis (RA), typified by inflammatory cell infiltration and joint damage. This study explores the therapeutic efficacy of HU-308, a CB2 receptor-specific agonist, on inflammation and immune balance in AIA.

**Methods:**

AIA was induced in mice by CFA injection. AIA mice were treated with HU-308 or vehicle, and effects on paw swelling, spleen index, histopathology, and immune cell profiles were evaluated. Flow cytometry, *in vitro* differentiation assays, and Western blot analysis were performed to examine Th17 and Treg cells, as well as signaling pathways involved in their differentiation.

**Results:**

HU-308 reduced paw swelling, lowered spleen index, and preserved joint integrity in AIA mice, mitigating inflammatory cell infiltration and bone erosion. Flow cytometry revealed that HU-308 restored the Th17/Treg imbalance in AIA, decreasing Th17 cell frequency and enhancing Treg cell infiltration. *In vitro* assays confirmed HU-308s role in promoting Treg differentiation and inhibiting Th17 polarization. Western blot analysis indicated that HU-308 modulated immune balance through the JAK/STAT5 and TGF-β/SMAD signaling pathways, increasing Foxp3 and TGF-β expression.

**Conclusion:**

HU-308 demonstrates significant anti-inflammatory effects in AIA by restoring Th17/Treg balance and reducing joint damage. The findings indicate that HU-308 holds potential as an immunomodulatory agent for RA, providing valuable insights into CB2-mediated therapeutic strategies for autoimmune diseases.

## 1 Introduction

Rheumatoid arthritis (RA) is a persistent autoimmune condition of uncertain etiology, characterized predominantly by inflammatory synovial pathology that leads to joint stiffness, swelling, pain, and deformities. This disorder is often accompanied by systemic manifestations, including weight loss, and fatigue, which significantly contribute to the high disability rate observed in affected individuals (Scott et al., 2010; Smolen et al., 2016). RA affects approximately 1% of the global population, with a higher prevalence observed in women. The chronic and progressive nature of RA imposes a considerable economic and social burden (Cross et al., 2014). Despite extensive research efforts, the intricate pathophysiology of RA remains only partially elucidated, with both genetic predispositions and environmental factors contributing to the onset and progression of the disease (Firestein and McInnes, 2017). Conventional anti-rheumatic pharmacotherapies, including nonsteroidal anti-inflammatory drugs (NSAIDs) and corticosteroids, are frequently employed in the management of RA. However, these treatments often exhibit limited efficacy and are associated with undesirable adverse effects, such as gastrointestinal complications, cardiovascular risks, and prolonged immune suppression (Singh et al., 2016; Yazdany et al., 2016).

In recent years, the advent of biologic therapies has markedly enhanced treatment outcomes for RA by specifically targeting cytokines and immune cell pathways implicated in the pathogenesis of the disease, such as tumor necrosis factor (TNF)-α, interleukin (IL)-6, and B cells (Feldmann and Maini, 2001; van Vollenhoven, 2009; Wu et al., 2021). Biologics have shown increased efficacy in alleviating RA symptoms and decelerating disease progression. Nevertheless, the inherent limitations associated with biologic therapies—namely, their substantial cost, the requirement for frequent injections, susceptibility to degradation, and potential adverse effects, including serious infections and an elevated risk of malignancies—underscore the persistent demand for the development of novel therapeutic agents for rheumatoid arthritis (Callegari et al., 2006; Bongartz et al., 2006; Singh et al., 2015).

Animal models, notably the adjuvant-induced arthritis (AIA) model, have demonstrated significant utility in RA research owing to their capacity to replicate the pathophysiological characteristics of RA (Hegen et al., 2008; Ahmad et al., 2006; Ahmad et al., 2014). The AIA model is initiated through the administration of complete Freund’s adjuvant (CFA), which elicits an immune response that closely resembles the synovial inflammation, joint destruction, and cellular immune imbalances observed in RA (Pearson, 1956; Sharma et al., 2004). This model is extensively employed in preclinical research to assess the efficacy and mechanisms of prospective RA therapies, encompassing immunomodulatory agents and innovative therapeutic strategies aimed at targeting inflammatory pathways (Hegen et al., 2008; Kollias et al., 2011).

The involvement of T cells in the pathogenesis of RA is extensively documented, with dysregulation among T cell subsets, particularly between T helper 17 (Th17) cells and regulatory T (Treg) cells, being pivotal in the progression of the disease (McInnes and Schett, 2011; Noack and Miossec, 2014; Weyand and Goronzy, 2021). Th17 cells, distinguished by their secretion of IL-17, are recognized for their pro-inflammatory properties, which facilitate osteoclast activation and contribute to joint destruction in RA (Yang et al., 2014; Yang et al., 2019). Conversely, Treg cells serve a protective function by suppressing autoimmune responses and maintaining immune homeostasis (Jiang et al., 2021). The equilibrium between Th17 and Treg cells is crucial for immune regulation, and disruption of this balance is associated with the pathogenesis of RA. In particular, an elevation in Th17 cells coupled with a reduction in Treg cells contributes to the inflammatory milieu characteristic of RA (Komatsu et al., 2014; Paradowska-Gorycka et al., 2020). Consequently, modulating the Th17/Treg balance has emerged as a promising therapeutic approach for re-establishing immune homeostasis in RA.

Cannabinoid receptors, particularly the CB2 receptor, are known for their immunomodulatory effects and have been investigated as potential therapeutic targets in various autoimmune and inflammatory diseases, including RA (Klein, 2005; Hua et al., 2020). Predominantly expressed on immune cells, CB2 plays a crucial role in modulating immune cell migration, cytokine production, and immune cell differentiation (Miller and Stella, 2008; Martinez-Aguilar et al., 2023). Prior research, including our investigations, has demonstrated that CB2 specific agonists possess therapeutic potential in the treatment of collagen-induced arthritis (CIA) and systemic sclerosis (SSc) through the modulation of T cell-mediated immune responses and the restoration of immune equilibrium (Gui et al., 2014; Tian et al., 2024). Furthermore, additional studies have indicated that activation of CB2 can affect the immune microenvironment by promoting anti-inflammatory responses and modifying the distribution of T cell subsets (Atalay et al., 2019; Merriam et al., 2008).

In this study, we investigate the effects of HU-308, a selective CB2 agonist, in the AIA model to assess its potential as a therapeutic agent for RA. The selective targeting of CB2 by HU-308 presents the advantage of minimizing central nervous system side effects that are commonly linked to the activation of CB1, thereby improving its safety profile for clinical applications (Maresz et al., 2007). We hypothesize that HU-308 can alleviate RA-like symptoms in AIA by modulating the Th17/Treg balance and reducing inflammatory infiltration in joint tissues. We aim to explore HU-308’s impact on joint pathology, immune cell profiles, and signaling pathways involved in T cell differentiation, with a particular focus on the JAK/STAT5 and TGF-β/SMAD pathways, which are critical for Th17 and Treg cell differentiation.

## 2 Methods

### 2.1 Mice

The mice were all of the C57BL/6J genetic background. They were housed under specific-pathogen-free (SPF) conditions, with environmental temperatures fluctuating around 21°C ± 1°C and humidity maintained at 60% ± 10%, under a 12/12-hour dark/light cycle, and provided unrestricted access to standard rodent chow. Mice were used for experiments starting at 6–8 weeks of age. The number of mice in each experimental group is indicated in the figure legends, with randomization and blinding strategies applied where possible. All animal experiments were conducted according to protocols approved by the Institutional Animal Care and Use Committee of Shanghai Jiao Tong University School of Medicine.

### 2.2 Induction of adjuvant-induced arthritis (AIA)

After anesthesia, mice were injected with 20 µL of 5 mg/mL Complete Freund’s Adjuvant (CFA) containing hot-killed *Mycobacterium tuberculosis* into the footpad under sterile conditions. This model has been widely utilized as an experimental framework for studying RA (Ahmad et al., 2006; Ahmad et al., 2014). The control group received an equivalent volume of saline in a similar manner. To evaluate the effects of HU-308 on the AIA mice, a dose of 1 mg/kg HU-308 (Tocris Bioscience, 3088) was selected based on prior studies and administered intraperitoneally, while the normal control group (control) and arthritis model group (AIA) received an equivalent volume of vehicle (4% DMSO, 1% tween-80, saline) (Tian et al., 2024; Sophocleous et al., 2015; Gui et al., 2015). The treatment regimen was administered once daily over a period of three weeks. Footpad thickness was assessed using a digital caliper at a consistent anatomical location, initially marked on day one, with subsequent measurements systematically recorded on days 1, 3, 7, 10, 14, and 21. Two independent, blinded researchers conducted triplicate measurements per time point to ensure accuracy and reduce bias. Upon completion of the three-week treatment period, spleen and ankle joint samples were harvested from each experimental group for subsequent flow cytometry staining and histopathological analysis.

### 2.3 Histological analysis

After 3 weeks of HU-308 treatment, the ankle joints of the mice were excised and subsequently immersed in a fixative solution comprising 10% formalin for a duration of 48 h. The samples underwent decalcification utilizing EDTA solution in 5% formic acid, after which they were subjected to dehydration and embedded in paraffin. The paraffin-embedded joint samples were sectioned using a microtome with a section thickness of 5 μm for detailed observation. The sections were stained with hematoxylin and eosin (H&E) to observe changes in joint tissue structure, such as synovial hyperplasia, cartilage destruction, and inflammatory cell infiltration.

### 2.4 Flow cytometry and cytokines production analysis

Flow cytometry data were collected using the LSRII (BD) flow cytometer and analyzed with FlowJo software. Single-cell suspensions derived from spleens were stained with Fixable Viability Dye eFluor 780 to exclude dead cells. For surface marker staining, cells were incubated on ice for 30 min with specific antibodies in FACS buffer (PBS containing 2% FBS and 2 mM EDTA). Intracellular staining was performed after fixing and permeabilizing the cells using BD Biosciences reagents. Cytokine expression was assessed by stimulating cells for 5 h in complete culture medium with phorbol 12-myristate 13-acetate (Sigma, P1585, 50 ng/mL), ionomycin (Sigma, I3909, 1 μM), and Golgi Stop (BD Biosciences, 554724), followed by staining with cytokine-specific antibodies. A list of antibodies for flow cytometry analysis is provided in Supplementary Table S1.

### 2.5 Isolation of murine T cell subsets and *in vitro* T cell differentiation

Lymphocytes were harvested from the spleen and peripheral lymph nodes, followed by the isolation of CD4^+^ T cells utilizing CD4(L3T4) Microbeads (Miltenyi Biotec, 130–049–201). Subsequently, naïve CD4^+^ T cells (characterized as CD4^+^CD25^−^CD44^−^CD62L+) were further purified using a BD FACS ARIA II or III cell sorter (BD Biosciences). The isolated T cells were then cultured in RPMI 1640 medium (Gibco, C22400500) supplemented with 10% fetal bovine serum (FBS, Gibco, 10100147), 1% GlutaMAX (Gibco, 35050061), 1% Sodium Pyruvate (Gibco, 11360070), 1% MEM Non-Essential Amino Acids Solution (Gibco, 11140050) and 1% Penicillin–Streptomycin. For *in vitro* differentiation, naïve CD4^+^ T cells were stimulated with 2 μg/mL plate-coated anti-CD3 (Bio-Xcell, BE0001-1) and 2 μg/mL soluble anti-CD28 (Bio-Xcell, BE0015-1) in the presence of various cytokines. iTreg differentiation was achieved with 50 U/ml IL2 and 0.25 ng/mL TGFβ (R&D Biotec, 7666-MB-005/CF). Th17 polarization was induced with 2 ng/mL TGFβ, 20 ng/mL IL6 (R&D Biotec, 406-ML-005/CF), 10 μg/mL anti-mouse IFNγ (Biolegend, 505710), and 10 μg/mL anti-mouse IL4 (Biolegend, 504135) in complete IMDM medium (Gibco, C12440500BT). Cells were collected at designated times post-differentiation for evaluation of differentiation efficiency via intracellular staining and flow cytometry.

### 2.6 *In vitro* suppression assay

CD4^+^ effector T cells (CD4^+^CD25^−^, Teff) were isolated and labeled with 2.5 μM CellTraceTM Violet (Invitrogen, #C34557) by incubation at 37°C for 15 min, followed by thorough washing to remove excess dye. Treg cells were then prepared at varying concentrations and cocultured with labeled Teff cells in a U-bottom 96-well plate to facilitate cell-to-cell interaction. Dynabeads Mouse T-activator CD3/CD28 (Teff cells:beads = 4:1) were added to each well to provide consistent stimulation. The cocultures were set up at Treg ratios of 1:1, 1:2, 1:4, 1:8, and 1:16, allowing for a gradient of Treg-mediated suppression levels. The cells were incubated for 84 h at 37°C in a humidified incubator with 5% CO_2_. Following incubation, the cells were stained with Viability Dye eFluor 780 to assess cell viability, ensuring that only live cells were included in the proliferation analysis. Flow cytometry was performed on a BD LSRFortessa X-20 to measure Teff cell proliferation. Proliferation was assessed by dilution of the CellTraceTM Violet dye, allowing quantification of cell division events. Data were analyzed to calculate the percentage of suppressed Teff cells in each condition, providing insights into the suppressive capacity of Treg cells at each ratio.

### 2.7 Western blot analysis

Cells were lysed on ice for 45 min in RIPA buffer supplemented with 1 mM PMSF, 1 mM Na3VO4, 1 mM NaF, and a protease inhibitor cocktail (Sigma). Lysates were clarified by centrifugation at 12,000 rpm for 15 min at 4°C, and the protein concentration of the supernatants was determined using the BCA Protein Assay Kit. Equal amounts of protein were loaded and separated by SDS-PAGE and subsequently transferred onto PVDF membranes (Millipore). Membranes were blocked with 5% non-fat milk in TBST (Tris-buffered saline with 0.1% Tween-20) for 1 h at room temperature to prevent non-specific binding. After blocking, membranes were incubated overnight at 4°C with primary antibodies (listed in Supplementary Table S2) diluted in TBST with 5% BSA. Following primary antibody incubation, the membranes were washed with TBST and incubated with HRP-conjugated secondary antibodies for 1 h at room temperature. Protein bands were visualized using an enhanced chemiluminescent (ECL) solution (Millipore) and imaged with a digital imaging system.

### 2.8 RNA isolation, reverse-transcription and real-time PCR

Total RNA was extracted using TRIzol reagent (Invitrogen, 15596018) following the manufacturer’s protocol. After extraction, RNA was quantified and assessed for purity using a NanoDrop spectrophotometer (Thermo Fisher Scientific). Complementary DNA (cDNA) was synthesized from 1 µg of total RNA using the HiScript II reverse transcriptase kit (Vazyme, R323-01) in a reaction volume of 20 µL according to the kit’s instructions. Quantitative PCR (qPCR) was performed using SYBR Green dye (Vazyme, Q711-02) on a ViiA7 PCR system (Applied Biosystems) in 384-well plates, with each reaction run in triplicate to ensure accuracy. The qPCR cycling conditions included an initial denaturation at 95°C for 10 min, followed by 40 cycles of 95°C for 15 s and 60°C for 1 min. Melt curve analysis was performed at the end of each qPCR run to confirm specificity of the amplification. Actin was used as the internal reference gene to normalize target gene expression levels. Relative expression levels were calculated using the 2 ^(-ΔΔCt)^ method. A list of primary primers used for the qPCR analysis is provided in Supplementary Table S3.

### 2.9 Statistical analysis

Data in this study are presented as means ± standard deviation (SD). Statistical analyses were conducted using GraphPad Prism 9.0. To compare differences between two independent groups, a 2-tailed Student’s t-test was performed, assuming normality of data distribution and equal variances. For comparisons among multiple groups, one-way analysis of variance (ANOVA) was applied. Normality of data distribution was assessed using the Shapiro-Wilk test, and homogeneity of variances was verified with Levene’s test. A significance level of p < 0.05 was set for all statistical tests. Levels of significance were denoted as follows: *p < 0.05, **p < 0.01, ***p < 0.001, ****p < 0.0001, with ns indicating non-significance.

## 3 Results

### 3.1 The CB2-specific agonist HU-308 can alleviate the severity of AIA

**FIGURE 1:**
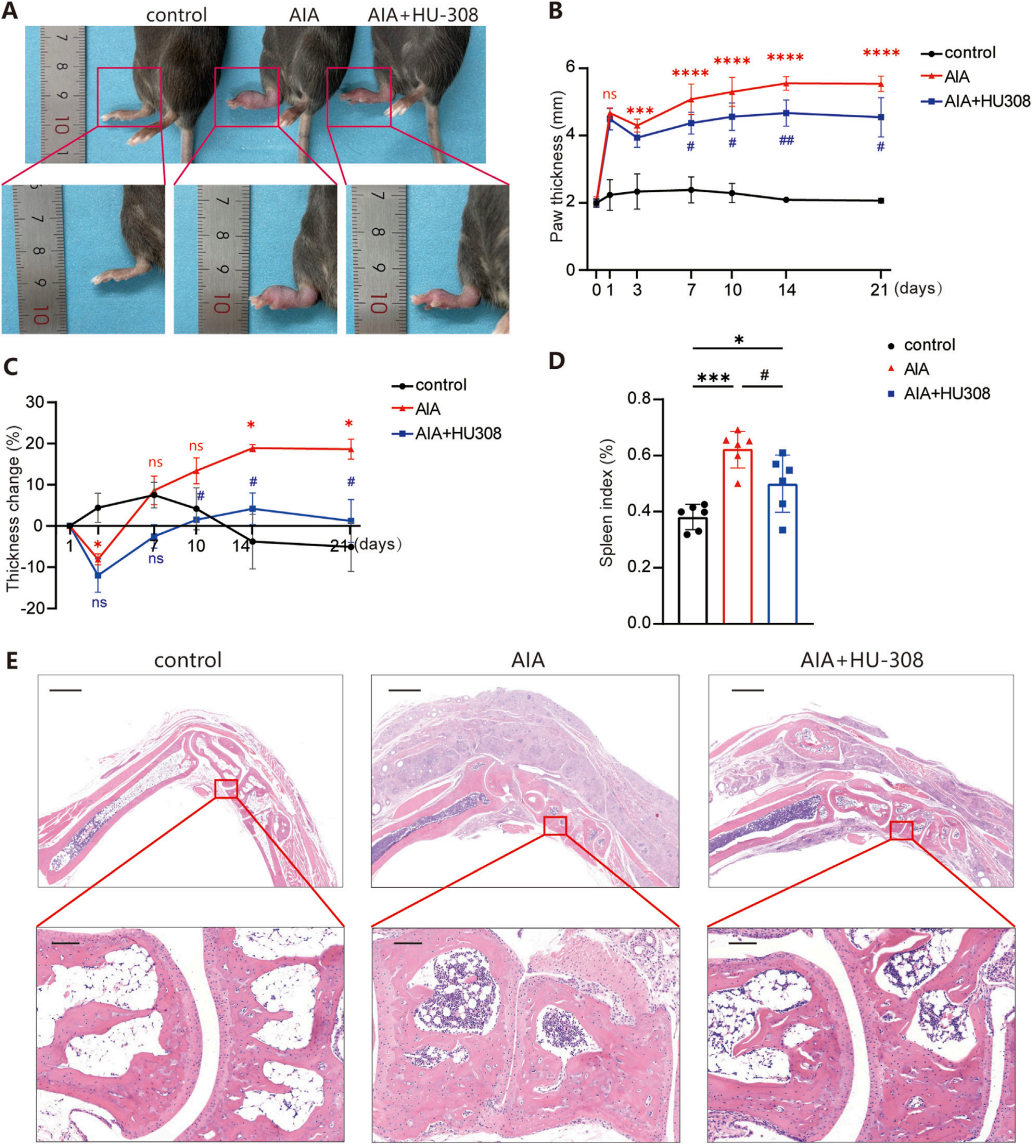
CB2 specific agonist HU-308 ameliorates adjuvant-induced arthritis (AIA) **(A)** Representative photographs of the paws from indicated mice. **(B)** The variation tendency of paw thickness across the different treatment groups. **(C)** Changes in paw thickness relative to the first day. **(D)** Spleen index (defined as the ratio of spleen mass to body mass) for each group of mice. **(E)** Representative histological images of hematoxylin and eosin (H&E) staining for the paws. (Horizontal scale bars = 1 mm in the lower magnification images; 100 µm in the higher magnification images). All data represent the mean ± SD, n = 5. Significance between every two groups was calculated using unpaired two-tailed Student’s t-test. *p < 0.05, ***p < 0.001 and ****p < 0.0001 compared to control group; #p < 0.05, ##p < 0.01 compared to AIA group. ns, not significant.

To evaluate the potential therapeutic effects of the highly selective CB2 receptor agonist HU-308 in mice with AIA, we established an AIA model by administering complete Freund’s adjuvant (CFA) into the mice’s paws, following protocols outlined in prior studies (Ahmad et al., 2006; Ahmad et al., 2014). Following induction, mice showed paw swelling and limited mobility. Treatment with HU-308 notably alleviated the paw swelling (Figure 1A). Quantitative analysis of paw thickness indicated that swelling in AIA mice peaked on the first day following CFA induction, partially subsided within three days, and then gradually worsened. However, HU-308 treatment significantly reduced paw thickness in AIA mice (Figure 1B). In comparison to the initial onset of symptoms, AIA mice treated with HU-308 exhibited improved paw recovery and a more rapid overall amelioration of symptoms (Figure 1C). Subsequently, we analyzed the spleen index across the treatment groups and observed a significant reduction in the spleen index of HU-308-treated AIA mice. This observation indicates that HU-308 may exert a beneficial effect on immune function and contribute to the maintenance of systemic immune homeostasis (Figure 1D). Histopathological examination of ankle joints in untreated mice with AIA demonstrated significant infiltration of inflammatory cells, predominantly lymphocytes and plasma cells, characteristic of RA, as well as bone erosion. Conversely, joint sections from the control group exhibited normal joint spaces and healthy tissue architecture. AIA mice treated with HU-308 showed a marked reduction in inflammatory cell infiltration, preservation of normal joint spacing, and maintenance of intact bone structure (Figure 1E). In our study, the safety of HU-308 was rigorously assessed through daily general health observations, regular body weight measurements, and histopathological examinations of major organs. No significant changes in mouse behavior, weight, or organ pathology were detected in HU-308-treated groups compared to controls, indicating a favorable safety profile (Supplementary Figures S1A, B). In summary, the findings indicate that HU-308 significantly alleviates inflammatory infiltration and joint tissue damage, consequently reducing swelling and enhancing mobility.

### 3.2 The CB2-specific agonist HU-308 effectively restores the Th17/Treg imbalance in AIA mice

To further investigate the mechanism by which HU-308 suppresses joint inflammation, immune cells were isolated from the spleens of mice in each experimental group, and alterations in the proportions of immune cell subpopulations were assessed using flow cytometry (Supplementary Figure S2A). In the splenic tissue of AIA mice, there was a reduction in the proportions of B cells and CD8^+^ T cells, while an increase in CD4^+^ T cells was observed, indicating that CD4^+^ T cells may play a pivotal role in the pathogenesis of AIA (Supplementary Figure S2B). Notably, the proportion of activated CD4^+^ T cells was significantly increased in AIA mice, underscoring the pathogenic role of these cells. However, the HU-308-treatment group exhibited the levels of both total and activated CD4^+^ T cells were similar to those observed in the control group (Supplementary Figures S2B, C). These findings suggest that HU-308 may primarily modulate CD4^+^ T cell differentiation to reshape the immune microenvironment in AIA mice, thereby reducing joint inflammation.

**FIGURE 2:**
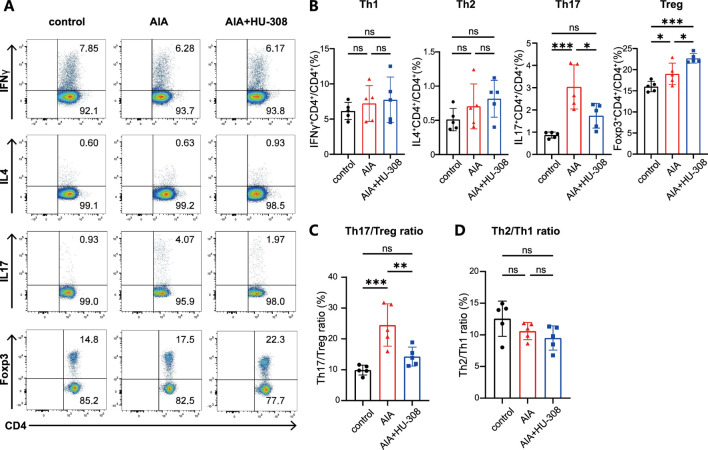
CB2 agonist HU-308 restores the Th17/Treg balance in AIA mice. **(A)** Representative flow cytometric plots illustrating the frequencies of Th1, Th2, Th17, and Treg cell populations in spleens. The numbers within the plots indicate the frequencies of cells present in each gate. **(B–D)** Summary plot showing frequencies of **(B)** Th1, Th2, Th17, Treg cells, **(C)** the Th2/Th1 ratio, and **(D)** the Th17/Treg ratio in spleens (n = 5). Data shown are representative of there independent experiments with similar results. Data are represented as the mean ± SD. *P < 0.05; **P < 0.01 and ***P < 0.001 by 2-tailed Student’s t-test. ns, not significant.

Our study then focused on the changes in CD4^+^ T cell subsets in the spleens of mice (Figure 2A). Flow cytometry analysis showed that, compared to control mice, AIA mice had a higher frequency of Th17 cells. However, HU-308 treatment reduced Th17 cell levels to the normal level of the control group (Figure 2B). Interestingly, Treg cells, known for their immunosuppressive function, also significantly increased in AIA mice, likely due to the overall rise in CD4^+^ T cells. Surprisingly, the administration of HU-308 further enhanced Treg cell infiltration in AIA mice (Figure 2B). Consequently, the Th17/Treg ratio was significantly disrupted in AIA mice, which was restored to normal by HU-308 treatment (Figure 2C). This finding elucidates the principal mechanism through which HU-308 mitigates AIA. In contrast, the proportions of Th1 and Th2 cells, as well as the Th1/Th2 ratio, remained stable in AIA mice (Figures 2B, D). These observations suggest that the progression of AIA is more reliant on the dysregulation of the Th17/Treg ratio, which can be effectively rectified by HU-308.

### 3.3 HU-308 can modulate Th17 and Treg cell differentiation *in vitro*

**FIGURE 3:**
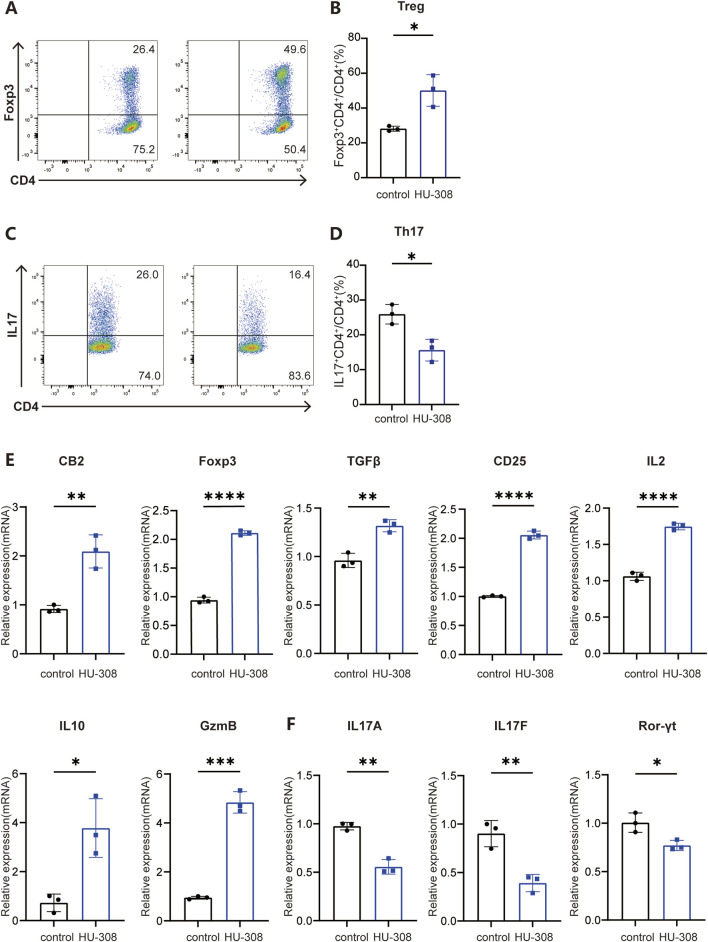
CB2 agonist HU-308 promotes the differentiation of Treg cells while impeding the differentiation of Th17 cells *in vitro*. Naïve CD4^+^ T cells were isolated from WT mice and cultured under stimulation with anti-CD3/CD28 antibodies in the presence of either Treg-polarizing conditions (IL-2 and TGF-β) or Th17-polarizing conditions (TGF-β, IL-6, anti-IL-4, and anti-IFN-γ). Cells were harvested at 24h for qPCR detection and at 72h for flow cytometry analysis. **(A)** Representative flow cytometric plots showing frequencies of Foxp3+ Treg cells under conditions of Treg differentiation, both in the presence and absence of 5 µM HU-308. The analysis was conducted on live CD4^+^ T cells, with the numerical values in the plots indicating the frequencies of cells within each specified gate. **(B)** Summary plots showing frequencies of Foxp3+ Treg cells with or without HU308 stimulation (n = 3). **(C)** Representative flow cytometric plots showing frequencies of IL17+ CD4^+^ T cells under the condition of Th17 differentiation with or without HU-308. Cells were gated on live CD4^+^ T cells. The numbers in the plots represent the frequencies of cells within each gate. **(D)** Summary plots showing frequencies of IL17+ CD4^+^ T cells with or without HU-308 stimulation (n = 3). **(E)** CB2, Foxp3, TGFβ, CD25, IL2, IL10 and GzmB CB2 gene expression normalized to Actin was evaluated by qPCR after 24h of differentiation of naive CD4^+^ T cells under Treg conditions (n = 3). **(F)** IL17A, IL17F and Ror-γt gene expression normalized to Actin was evaluated by qPCR after 24h of differentiation of naive CD4^+^ T cells under Th17 conditions (n = 3). Data are represented as the mean ± SD. *P < 0.05; **P < 0.01; ***P < 0.001 and ****P < 0.0001 by 2-tailed Student’s t-test.

**FIGURE 4:**
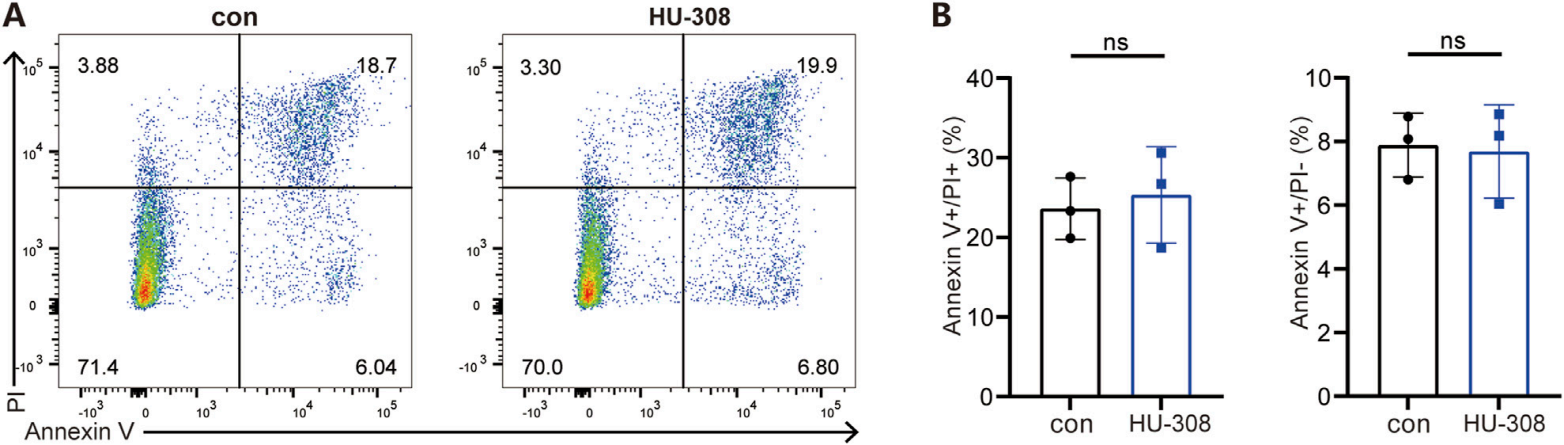
HU-308 has no impact on the apoptosis of CD4^+^ T cells. Naïve CD4^+^ T cells were isolated from WT mice and stimulated with anti-CD3/CD28 antibodies. Cells were harvested at 72h for Annexin V/PI staining and flow cytometry analysis with or without 5 µM HU-308. **(A)** Annexin V/PI staining and flow cytometry were used to investigate the effects of HU-308 on the apoptosis of CD4^+^ T cells. **(B)** The proportion of Annexin V-positive and PI-positive (Annexin V+/PI+) and Annexin V-positive and PI-negative (Annexin V+/PI−) cells were summarized. Data are shown as the mean ± SD. of three biological replicates by 2-tailed Student’s t-test.

To further investigate the direct effects of HU-308 on Th17 and Treg cells, we conducted *in vitro* cell differentiation experiments with or without HU-308 stimulation. Cells were collected and stained for flow cytometry to assess the differentiation efficiency of different subtypes. Flow cytometry results showed that HU-308 significantly promoted Treg cell differentiation *in vitro* (Figures 3A, B) while inhibiting Th17 cell polarization (Figures 3C, D). Additionally, qPCR analysis during naive CD4^+^ T cell differentiation revealed that HU-308 induced an upregulated expression of CB2 receptors after activating the CB2 receptor, potentially enhancing activation effects of HU-308. The transcription factor Foxp3, specific to Treg cells (Sakaguchi, 2005), exhibited a significant upregulation following HU-308 treatment. Similarly, another crucial signaling molecule, TGF-β, which plays a pivotal role in Treg differentiation (Josefowicz et al., 2012), also showed an elevation post HU-308 stimulation. Furthermore, additional functional markers associated with Tregs (Josefowicz et al., 2012), including CD25, IL-2, IL-10, and Granzyme B (GzmB), were upregulated (Figure 3E). Contrary to expectations, the suppressive function of Treg cells did not demonstrate a significant change after HU-308 treatment. This observation suggests that the effect of HU-308 on Treg cells is predominantly associated with promoting Treg cell differentiation and increasing their numbers within tissues, rather than enhancing their suppressive function (Supplementary Figures S3A, B). Conversely, the Th17-specific transcription factor Ror-γt, along with cytokines IL-17A and IL-17F (Iwakura et al., 2011), exhibited a significant reduction following HU-308 stimulation, aligning with HU-308’s inhibitory effect on Th17 differentiation (Figure 3F). To further address the potential effects of HU-308, we have evaluated its impact on cellular apoptosis. Our findings indicated that HU-308 does not affect apoptosis of CD4^+^ T cells, suggesting that its observed effects are mediated through immunomodulatory mechanisms rather than cytotoxicity (Figures 4A, B). Collectively, these findings suggest that HU-308 is capable of inhibiting Th17 cell differentiation and IL-17 production while simultaneously promoting Treg cell differentiation *in vitro*.

### 3.4 HU-308 regulates the Th17/Treg balance through JAK/STAT5 and TGF-β/SMAD signaling

**FIGURE 5:**
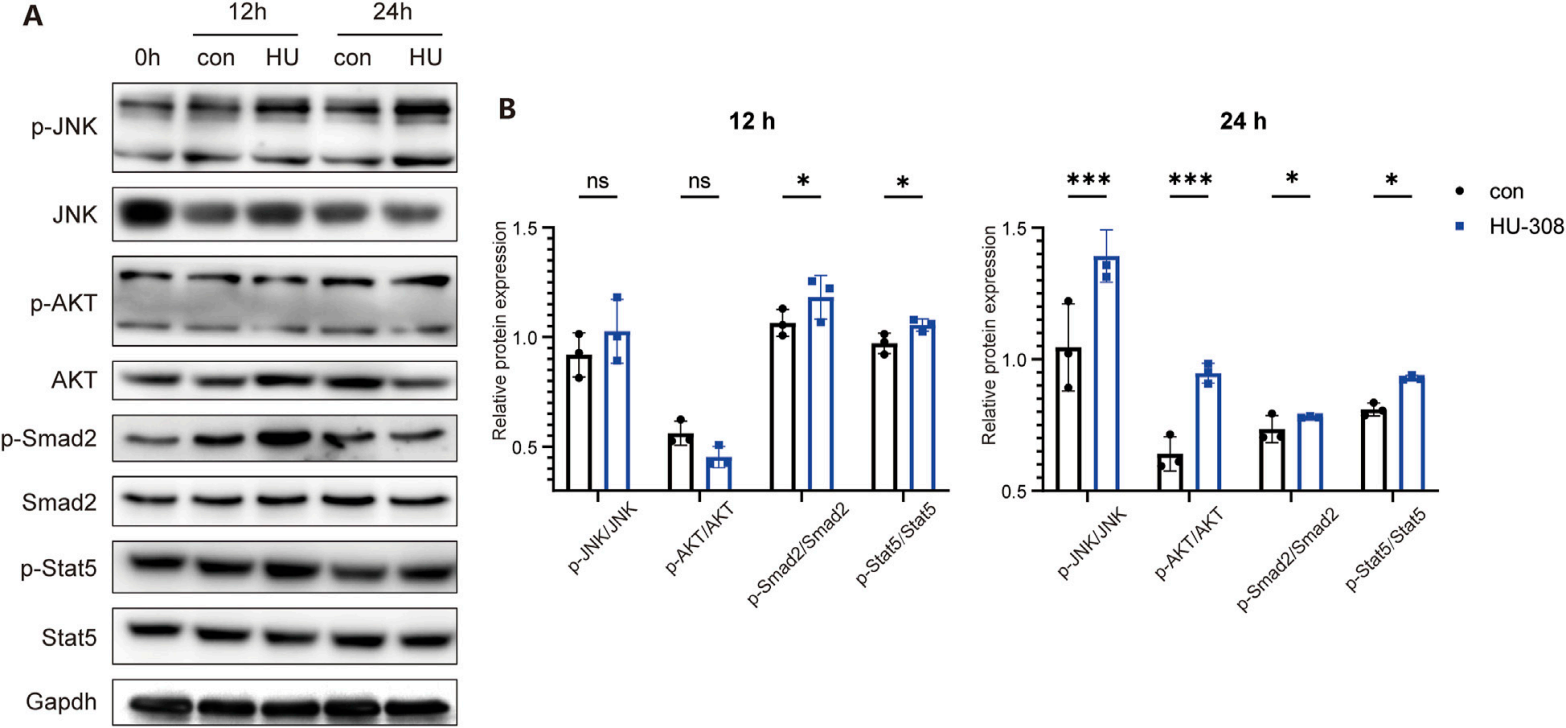
CB2 agonist HU-308 regulates the Th17/Treg differentiation through JAK/STAT5 and TGF-β/SMAD signaling. Naive CD4^+^ T cells were sorted and cultured under stimulation conditions using anti-CD3/CD28 antibodies and 20U IL2, with or without the addition of HU-308. Cells were harvested at 12h and 24h respectively. **(A)** p-JNK/JNK, p-AKT/AKT, p-Smad2/Smad2, p-Stat5/Stat5 and Gapdh changes were detected by Western blot. **(B)** The semi-quantitative analysis for Western blot. Data shown are representative of there independent experiments with similar results. Data are represented as the mean ± SD. *P < 0.05 and ***P < 0.001 by two way ANOVA. ns, not significant.

As a significant component of the GPCRs family, upon activation, CB2 is capable of mediating the conventional downstream signaling pathways characteristic of GPCRs, such as the phosphorylation of AKT (p-AKT/AKT) (Dhopeshwarkar and Mackie, 2014). Furthermore, CB2 activation is linked to additional signaling pathways, including JAK/STAT pathway (Xiong et al., 2022). In the context of Treg and Th17 cell differentiation, multiple signaling pathways interact in a coordinated manner (Zhang et al., 2021). Notably, TGF-β pathway is a common requisite for the differentiation processes of both Th17 and Treg cells. TGF-β initially interacts with TGF-βR, thereby initiating the activation of downstream SMAD transcription factors, specifically SMAD2 and SMAD3, in conjunction with the common mediator SMAD4. The resultant activated SMAD complex translocates into the nucleus, where it modulates the transcription of RORγt and Foxp3 (Wang et al., 2023; Schmierer and Hill, 2007; Derynck and Zhang, 2003). The expression of RORγt facilitates the differentiation of CD4^+^ T cells into the Th17 lineage, whereas the expression of Foxp3 directs the differentiation of CD4^+^ T cells towards the Treg lineage (Kumar et al., 2021; Chen et al., 2011). Another indispensable signal in Treg differentiation, IL-2, in conjunction with TGF-β, promotes the tyrosine phosphorylation of STAT5, which in turn enhances Foxp3 synthesis, ultimately polarizing naive CD4^+^ T cells towards Treg (Josefowicz et al., 2012). Therefore, we first examined the traditional downstream signaling pathways of GPCRs. Western blot results demonstrated that stimulation with HU-308 led to the activation of the p-JNK/JNK and p-AKT/AKT signaling pathways in naive CD4^+^ T cells (Figures 5A, B). Concurrently, there was a significant upregulation of the classical downstream TGF-β signaling molecule p-Smad2/Smad2. Additionally, the pivotal signaling pathway p-Stat5/Stat5, which is associated with Treg differentiation, was also markedly activated. The findings indicate that HU-308 modulates the Th17/Treg balance via the JAK/STAT5 and TGF-β/SMAD signaling pathways.

## 4 Discussion

This study highlights the therapeutic potential of HU-308, a highly selective CB2 agonist, in the context of AIA, a well-established model for RA. By reducing inflammation, restoring Th17/Treg balance, and preserving joint integrity, HU-308 demonstrates significant immunomodulatory effects that align with previous research on CB2 receptor agonists while also introducing novel insights into their mechanism of action.

Our findings support prior research indicating that CB2 activation can effectively modulate immune responses and attenuate inflammation in autoimmune diseases. Previous studies have demonstrated that CB2 agonists, such as HU-308, play an anti-inflammatory role by inhibiting cytokine production and immune cell migration (Pacher and Mechoulam, 2011; Turcotte et al., 2016). Specifically, studies on CIA and other autoimmune conditions have shown that CB2 agonists can reduce inflammatory responses in joint tissues, a conclusion consistent with our observations in AIA (Gui et al., 2014). Notably, HU-308 treatment reduced paw swelling and decreased inflammatory cell infiltration in joint tissues in murine models of arthritis where CB2 activation limited immune-mediated joint damage.

In addition, our study regards the role of Th17 and Treg cells in RA pathogenesis and the therapeutic effects of CB2 modulation in restoring their balance. Th17 cells are known for their pathogenic effects in RA, mainly through IL-17 production, which drives inflammatory cascades and joint damage. Conversely, Treg cells play a protective role by suppressing inflammation and promoting immune tolerance (Kumar et al., 2021; Knochelmann et al., 2018). Our results align with existing studies that suggest CB2 activation can normalize the Th17/Treg imbalance by increasing Treg cell infiltration and reducing Th17 cell frequency. These findings indicate that HU-308 not only suppresses inflammatory pathways but also actively reshapes the immune microenvironment in a manner conducive to RA management.

In our study, we have utilized HU-308, a highly selective agonist for CB2, to explore its therapeutic potential in the AIA model. The specificity of HU-308 for CB2 has been previously established through molecular dynamics (MD) and metadynamics (metaMD) simulations, which have been critical in elucidating the interaction dynamics between HU-308 and CB2, demonstrating a high affinity and selective receptor engagement (Hua et al., 2020). Moreover, our previous work in a systemic sclerosis (SSc) model provides additional context, where HU-308’s effects were markedly diminished in the absence of CB2, underscoring its receptor-specific activity (Tian et al., 2024). These observations are particularly relevant considering reports of other CB2 agonists, such as JWH-015, which have demonstrated alternative pathways of action, including interactions with glucocorticoid receptors, potentially complicating the interpretation of results solely based on receptor pharmacology (Fechtner et al., 2019a). Thus, the high specificity of HU-308 not only enhances our understanding of CB2 pharmacodynamics but also highlights the therapeutic potential of targeting this pathway in autoimmune diseases like RA.

While previous studies on CB2 agonists indicated a general reduction in T cell populations (Gaffal et al., 2020; Graham et al., 2010), we observed that HU-308 did not significantly decrease the total or activated CD4^+^ T cell population. Instead, HU-308 primarily altered the differentiation of CD4^+^ T cells into specific subsets, notably reducing the proportion of Th17 cells and enhancing Treg cell differentiation. This finding suggests that HU-308 exerts its immunomodulatory effects not by broadly suppressing immune activation but by selectively promoting immune homeostasis through targeted T cell modulation. Such results highlight a potential advantage of HU-308, as selective immune modulation may mitigate some of the side effects associated with broader immunosuppressive therapies. In our previous study, HU-308 ameliorated SSc by inhibiting Th2 cell differentiation in the spleen and lung tissues (Tian et al., 2024). However, in this study, HU-308 did not affect Th2 cells in the spleen but selectively targeted Th17 and Treg cells. This discrepancy may arise from the distinct immunopathological mechanisms between the AIA and SSc models, with the former primarily mimicking inflammatory processes in RA and the latter involving fibrosis, could account for the varied effects of HU-308 on T cell subsets. Furthermore, the context-dependent action of HU-308 may be influenced by the specific immune microenvironment and inflammatory state, which differ between the AIA and SSc models, potentially enhancing its influence on Th17 and Treg cells in AIA. These findings underscore the complexity of drug action in different disease contexts and highlight the need for tailored therapeutic strategies.

Moreover, in our *in vitro* studies, HU-308 was found to directly influence Treg and Th17 differentiation pathways. The CB2 agonist promoted Treg-specific markers such as Foxp3 and increased TGF-β signaling, which are critical for Treg differentiation. Simultaneously, HU-308 suppressed Th17-related markers, including Rorγt and IL-17A. While other studies have reported the immunosuppressive effects of CB2 agonists, direct *in vitro* evidence of their role in influencing T cell differentiation pathways is limited (Chen et al., 2022; Sarsembayeva et al., 2022; Leinwand et al., 2017). Thus, our study provides more mechanistic insights into how CB2 activation affects key signaling pathways involved in immune cell differentiation.

Additionally, we demonstrated that HU-308 activated the JAK/STAT5 and TGF-β/SMAD pathways, essential for Treg differentiation and Th17 inhibition. This mechanistic insight adds a new dimension to our understanding of how CB2 receptor activation impacts immune regulation beyond its known anti-inflammatory effects. While prior research has focused on the overall reduction of pro-inflammatory cytokines following CB2 activation, few studies have explored the specific intracellular signaling cascades that regulate T cell differentiation (Klein, 2005; Marcu et al., 2010).

Focusing on the AIA model, which replicates RA-like inflammation and immune dysregulation in a manner distinct from CIA and other autoimmune models (Hegen et al., 2008), allowed us to study HU-308s effects on a broader range of RA-like symptoms, including joint swelling, immune cell infiltration, and bone integrity. This approach helps establish HU-308 as a potential therapeutic option for RA by demonstrating efficacy in a model with distinct inflammatory and immune characteristics.

However, due to the technical challenges associated with the anatomical and physiological constraints of the AIA model, we were unable to directly assess the CD4^+^ T cells, Th17, and Treg populations within the synovial tissues. Similar difficulties have been reported by other research groups, which underscores the complexity of conducting such analyses in AIA models (Jin et al., 2024; Kong et al., 2023). Despite these challenges, we innovatively investigated the effects of HU-308 on systemic immune markers, such as the spleen index, which serves as an indicator of systemic immune regulation. The significant reduction in the spleen index of HU-308-treated AIA mice suggests that HU-308 may help restore systemic immune homeostasis, a feature not thoroughly explored in previous research focused primarily on local joint effects (Bryk and Starowicz, 2021; Fechtner et al., 2019b; Zhu et al., 2019). By examining both local and systemic outcomes, this study provides a more comprehensive assessment of HU-308s therapeutic potential in RA.

Despite the findings, our study is not without limitations. The efficacy of HU-308 observed in AIA mice may not directly translate to human RA patients, necessitating further validation in clinical trials. Although our study shows that HU-308 regulates Th17/Treg balance via the JAK/STAT5 and TGF-β/SMAD pathways, the underlying molecular mechanisms are likely more intricate. CB2 may engage other pathways not yet explored, warranting future research using gene editing or RNA interference to delineate CB2 agonist mechanisms in various cell types. Lastly, this study primarily focuses on HU-308’s short-term effects, lacking an assessment of long-term efficacy and potential adverse effects. Prolonged CB2 activation could pose immunosuppressive risks, necessitating further studies on long-term safety and immune responses.

## 5 Conclusion

Our study underscores the therapeutic potential of HU-308, a selective CB2 agonist, in mitigating AIA in mice, serving as a model for RA. Our findings reveal that HU-308 effectively alleviates AIA severity by modulating the Th17/Treg imbalance and targeting key signaling pathways involved in T cell differentiation, specifically JAK/STAT5 and TGF-β/SMAD. The ability of HU-308 to restore immune homeostasis and ameliorate joint pathology without broad immunosuppression suggests a nuanced approach to RA treatment. While these results are promising, further research is needed to translate these effects to human RA and to explore the long-term implications of CB2 activation. The current study provides a foundation for the development of HU-308 as a novel therapeutic strategy for RA, highlighting the importance of selective immune modulation in autoimmune disease management.

## Data Availability

The original contributions presented in the study are included in the article/Supplementary Material, further inquiries can be directed to the corresponding authors.
