# Supplementary material for: Cannabinoid receptor 2 selective agonist ameliorates adjuvant-induced arthritis by modulating the balance between Treg and Th17 cells
Source: Front Pharmacol. 2025 Jan 31;16:1532518. doi: 10.3389/fphar.2025.1532518 (PMC11825454; doi:10.3389/fphar.2025.1532518)
Supplement: Supplementary file 1 [file DataSheet1.docx]

**Supplementary materials**

**Supplementary Figure 1**


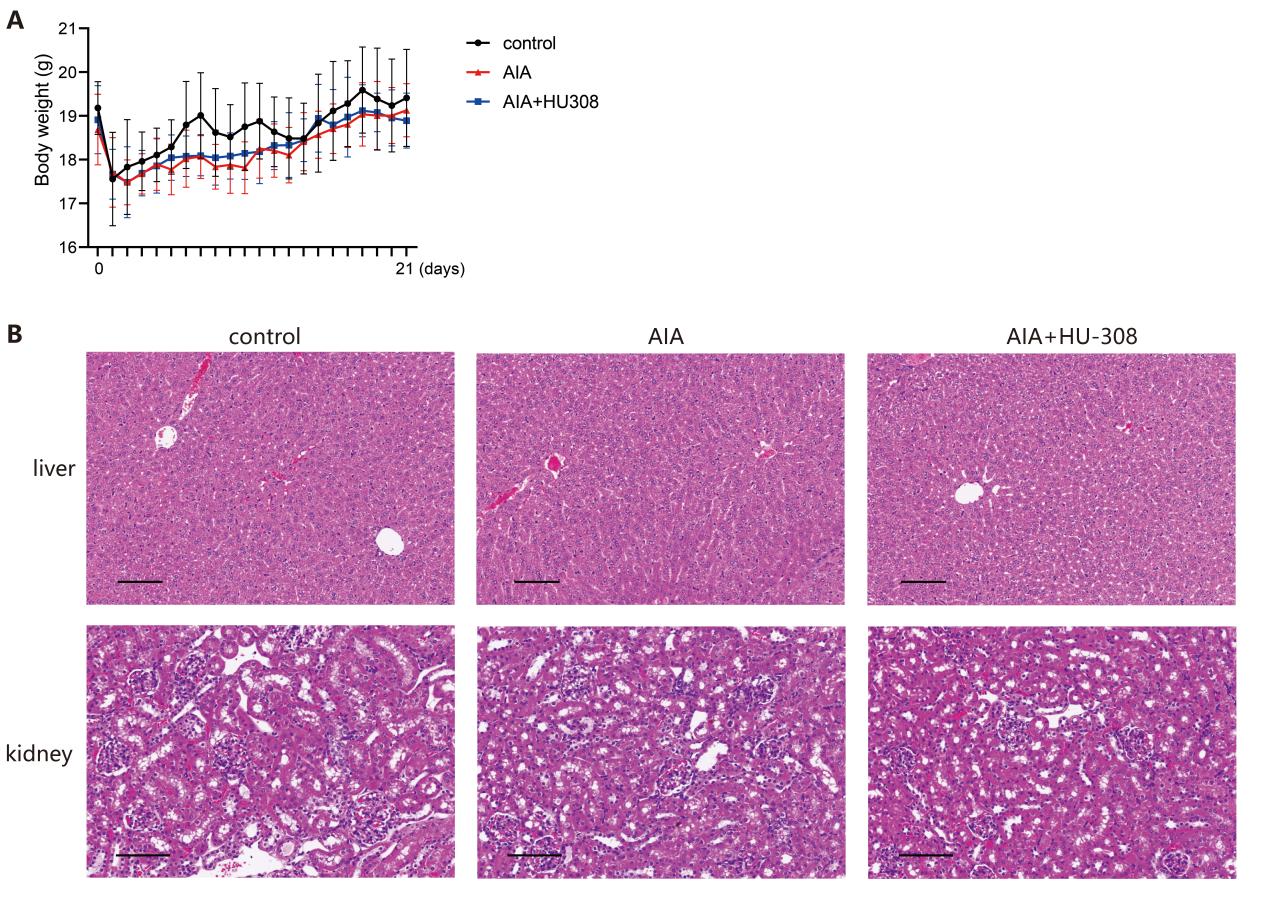


**Supplementary Figure 1 HU-308 does not affect mouse body weight or liver and kidney histology.** (A) Changes in body weight of mice in different groups during the treatment period with HU-308. The graph shows the average body weight±SEM for each group over the treatment course, indicating no significant weight loss or gain in any of the groups treated with HU-308 compared to not treated. (B) Hematoxylin and eosin (H&E) staining of liver and kidney sections from mice at the end of the HU-308 treatment. Images show representative histology from each group.

**Supplementary Figure 2**

**
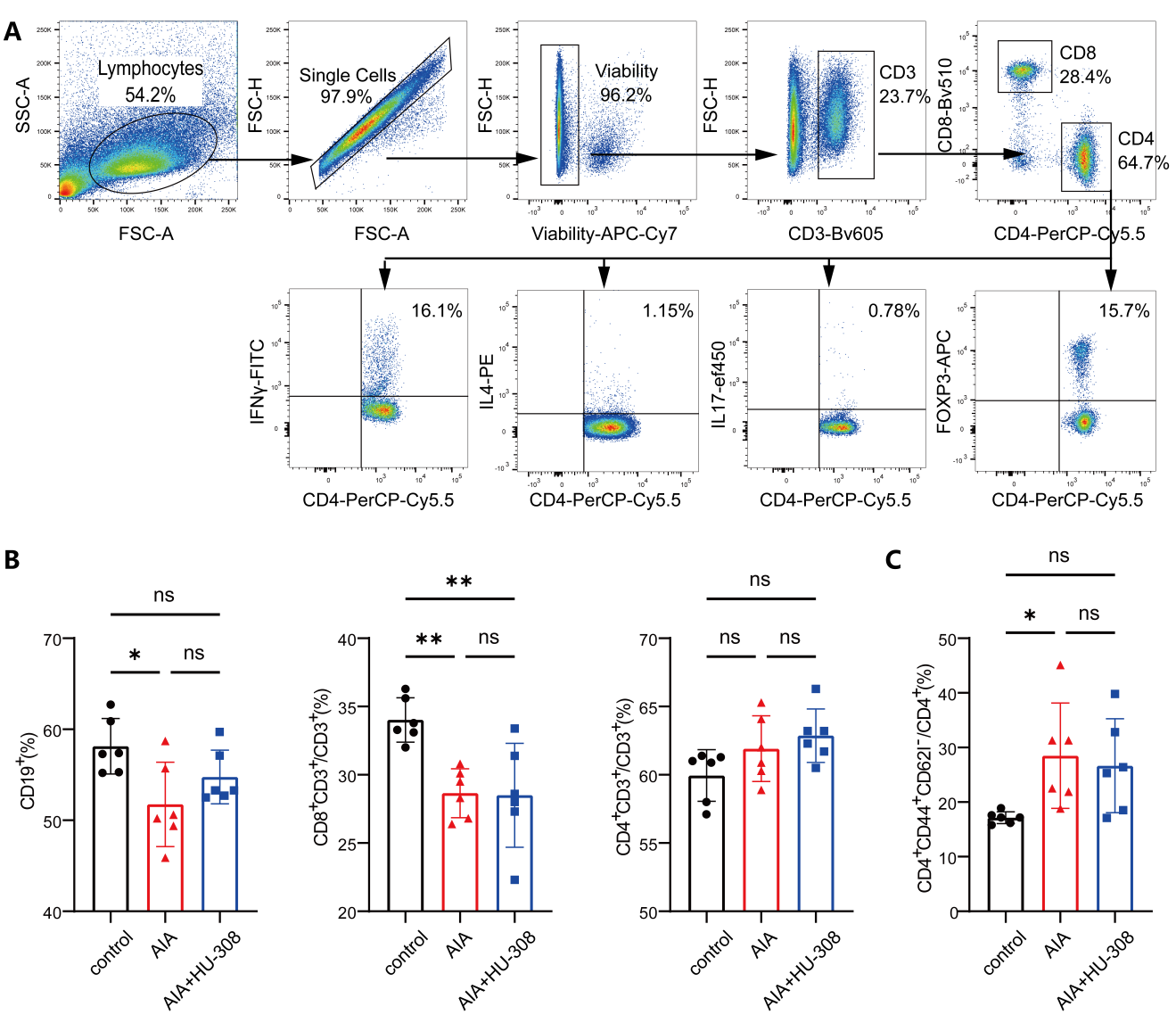
**

**Supplemental Figure 2 CB2 agonist HU-308 modulates the distribution of immune cell populations in mice with adjuvant-induced arthritis (AIA).** (A) Flow cytometry gating strategy for immune cells isolated from the spleen. (B) Summary plot showing frequencies of CD19+, CD8+ CD3+, CD4+ CD3+ and activated CD4+ T cells (CD4+ CD44+ CD62l- ) in the spleen of mice in each group (n=6). Data shown are representative of there independent experiments with similar results. Data are represented as the mean ± SD. *p<0.05 and **p<0.01 by 2-tailed Student’s t test. NS, not significant.

**Supplementary Figure 3**


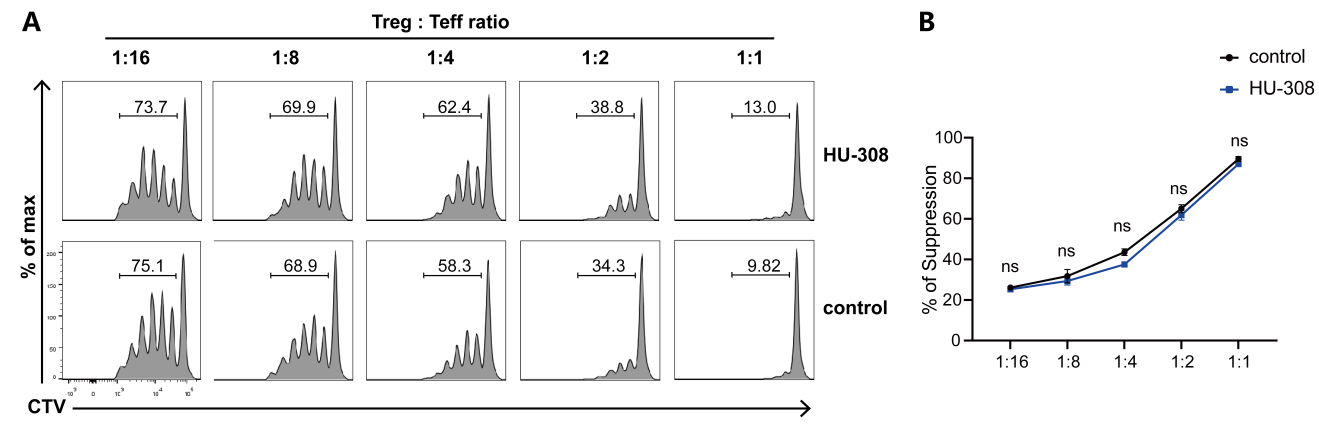


**Supplemental Figure 3 HU-308 had no impact on Treg cell function.** (A) *In vitro* suppression assay was conducted on mouse nTreg cells, with or without HU-308 stimulation. Mouse nTreg and CD4^+^ T effector cells (responder) were cocultured for 84h at varying ratios 1:1, 1:2, 1:4, 1:8 or 1:16) using anti-CD3/CD28 beads for activation. Proliferation of responder cells was tracked via CTV dye dilution. (B) Proportion of CD4^+^ T effector cell proliferation suppressed by nTreg cells. Data shown are representative of there independent experiments with similar results. Data are represented as the mean ± SD. The significances were measured by 2-tailed Student’s t test. NS, not significant.

**Supplementary Table 1:** Monoclonal antibodies used for flow cytometry.

| **Antibody name** | **Company** | **Catalog No.** |
| --- | --- | --- |
| Viability Dye eFluor 780 | eBioscience | 65-0865-14 |
| Anti-Mouse-CD3 Bv605 | BioLegend | 100237 |
| Anti-Mouse-CD4 FITC | eBioscience | 11-0041-85 |
| Anti-Mouse-CD4 PE | eBioscience | 12-0042-85 |
| Anti-Mouse-CD4 PercP-Cy^TM^5.5 | Tonbo bioscience | 65-0042-U100 |
| Anti-Mouse-CD8a Bv510 | BioLegend | 100752 |
| Anti-Mouse-CD19 PE | eBioscience | 12-0193-82 |
| Anti-Mouse-CD45 ef450 | eBioscience | 48-0454-82 |
| Anti-Mouse-CD44 PE | Miltenyi Biotec | 130-102-606 |
| Anti-Mouse-CD62L APC | Tonbo bioscience | 20-0621-U100 |
| Anti-Mouse-CD25 PE | eBioscience | 12-0251-82 |
| Anti-Mouse-IL4 PE | eBioscience | 12-7041-82 |
| Anti-Mouse-IFNγ FITC | Tonbo bioscience | 35-7311-U100 |
| Anti-Mouse-IL-17A ef450 | eBioscience | 48-7177-82 |
| Anti-Mouse-Foxp3 APC | eBioscience | 17-5773-82 |

**Supplementary Table 2:** Monoclonal antibodies used for Western blot (WB).

| **Antibody name** | **Company** | **Catalog No.** |
| --- | --- | --- |
| Phospho-SAPK/JNK (Thr183/Tyr185) Antibody | Cell Signaling Technology | 9251 |
| SAPK/JNK Antibody | Cell Signaling Technology | 9252 |
| Phospho-Akt (Ser473) (D9E) XP® Rabbit mAb | Cell Signaling Technology | 4060 |
| Akt Antibody | Cell Signaling Technology | 9272 |
| Phospho-SMAD2 (Ser465/Ser467) (E8F3R) Rabbit mAb (Biotinylated) | Cell Signaling Technology | 55041 |
| SMAD2 (D43B4) XP® Rabbit mAb | Cell Signaling Technology | 5339 |
| Phospho-Stat5 (Tyr694) (D47E7) XP® Rabbit mAb | Cell Signaling Technology | 4322 |
| Stat5 (D2O6Y) Rabbit mAb | Cell Signaling Technology | 94205 |
| GAPDH Monoclonal Antibody | Proteintech | 60004-1-Ig |

**Supplementary Table 3:** Real-Time Polymerase Chain Reaction (qPCR) Primers.

| **Gene** | **Forward (5’-3’)** | **Reverse (5’-3’)** |
| --- | --- | --- |
| Actin | TGGAATCCTGTGGCATCCATGAAAC | TAAAACGCAGCTCAGTAACAGTCCG |
| CB2 | CTCGTACCTGTTCATCAGCAGC | CAGCAGGAAGATAGCGTTGGAG |
| Foxp3 | GGCCCTTCTCCAGGACAGA | GCTGATCATGGCTGGGTTGT |
| TGFβ | CTCCCGTGGCTTCTAGTGC | GCCTTAGTTTGGACAGGATCTG |
| CD25 | GCGTTGCTTAGGAAACTCCTGG | GCATAGACTGTGTTGGCTTCTGC |
| IL2 | GCGGCATGTTCTGGATTTGACTC | CCACCACAGTTGCTGACTCATC |
| IL10 | GCTCTTACTGACTGGCATGAG | CGCAGCTCTAGGAGCATGTG |
| GzmB | CAGGAGAAGACCCAGCAAGTCA | CTCACAGCTCTAGTCCTCTTGG |
| IL17A | TCCAGAAGGCCCTCAGACTA | CTCGACCCTGAAAGTGAAGG |
| IL17F | CCCAGGGTCAGGAAGACA | CCGAAGGACCAGGATTTCT |
| Ror-γt | AACCAGGCATCCTGAACTTG  | CGTAGAAGGTCCTCCAGTCG |
